# The effect of *Lactiplantibacillus plantarum* probiotic supplement on rainbow trout challenged with *Aeromonas salmonicida*

**DOI:** 10.17221/101/2025-VETMED

**Published:** 2026-04-30

**Authors:** Ivana Mikulikova, Zuzana Lepkova, Hana Bandouchova, Jana Blahova, Ivana Papezikova, Hana Novotna, Ivona Toulova, Katerina Kobelkova, Klara Odehnalova, Eva Postulkova, Marija Radojicic, Jan Mares, Dagmar Mudronova, Miroslava Palikova

**Affiliations:** ^1^University of Veterinary Sciences Brno, Brno, Czech Republic; ^2^Mendel University in Brno, Brno, Czech Republic; ^3^Veterinary Research Institute, Brno, Czech Republic; ^4^Czech Academy of Sciences, Brno, Czech Republic; ^5^University of Veterinary Medicine and Pharmacy in Košice, Košice, Slovak Republic; Ivana Mikulikova and Zuzana Lepkova contributed equally to this work.

**Keywords:** furunculosis, gut microbiota, lactobacilli, *Oncorhynchus mykiss*

## Abstract

Two probiotic (*Lactiplantibacillus plantarum*) supplementation strategies (continuous and cyclic) were evaluated for their ability to enhance resistance of rainbow trout (*Oncorhynchus mykiss*) to *Aeromonas salmonicida* infection. Neither of these strategies improved post-challenge survival. Instead, cyclic administration resulted in a significantly higher mortality rate (73%) compared with continuous supplementation (52%) and the control group (46%). One week post-challenge, most haematological, plasma biochemical, and immune parameters showed no significant difference between treatments, though fish receiving cyclic supplementation did exhibit a reduced lymphocyte count. However, three weeks post-challenge, this same group showed a significant decrease in total phagocyte number and in the proportion of phagocytes within white blood cells. IgM concentrations were significantly lower in both probiotic-supplemented groups than in the control group. In the cyclic group, reductions in interleukin-10 and elevations in total protein levels were also observed. Microbiome analysis of gut content three weeks post-challenge revealed a marked decline in microbial diversity in both probiotic-treated groups. These findings indicate that, under the experimental conditions, probiotic supplementation did not provide protection against *A. salmonicida* infection and that cyclic administration may disrupt immune homeostasis and intestinal microbial stability, ultimately compromising host resilience.

Several approaches have been adopted to prevent and control bacterial infections in aquaculture as alternatives to antibiotic/chemotherapeutic treatment. These include vaccination, selective breeding, and the use of feed supplements containing prebiotics and/or probiotics (Hoseinifar et al. 2024). The antimicrobial properties of probiotics are mediated by the production of antimicrobial substances, the competitive exclusion of pathogens, and the enhancement of the host’s barrier function and immune system (Fijan 2023). The effectiveness of dietary probiotics will be influenced by factors such as the species and age of the fish, the dosage applied, and the feeding strategy employed, e.g., the timing of probiotic application. The most widely used aquacultural probiotics nowadays are the lactobacilli, which are Gram-positive, facultatively anaerobic, fermentative, non-spore-forming microorganisms. Of these, *Lactiplantibacillus plantarum* (previously known as *Lactobacillus plantarum*) has been reported to positively affect weight gain and feed conversion ratio in several fish species (e.g., Nguyen et al. 2019; Soltani et al. 2019). The autochthonous strain, *Lactiplantibacillus plantarum* R2 Biocenol^TM^ (CCM 8674), which was isolated from the intestines of healthy rainbow trout *Oncorhynchus mykiss*, has been shown to exhibit distinct probiotic potential in *in vitro* tests (Feckaninova et al. 2019). Pretreating rainbow trout intestinal cells in primoculture with products of *L. plantarum* R2 Biocenol^TM^ (CCM 8674) (identified as lactic acid, acetic acid, acetoacetic acid, succinic acid and formic acid) was found to reduce inflammation following *Aeromonas salmonicida* (CCM 1307) infection by decreasing the expression of pro-inflammatory cytokines (Cingelova Maruscakova et al. 2021).

*A.* *salmonicida* is one of the most relevant aquatic pathogens leading to significant economic losses in salmonid aquaculture due to mass morbidity and mortality of susceptible species. This Gram-negative bacterium is the causative agent of furunculosis, which may manifest in peracute, acute, or chronic form depending especially on the age of the fish (Menanteau-Ledouble et al. 2016; Jun et al. 2022).

Although vaccines are available, protection against furunculosis is often inconsistent, and outbreaks persist despite vaccination efforts. The potential adverse effects of vaccination must also be taken into consideration (Gudding and Van Muiswinkel 2013; Bogwald and Dalmo 2019).

This study builds upon a previous study by Mikulikova et al. (2025), which assessed the use of *L.* *plantarum* R2 Biocenol^TM^ (CCM 8674) as a feed supplement for rainbow trout. In brief, continuous supplementation increased the oxygen-carrying capacity of fish blood, and a similar positive effect on growth performance in juvenile fish was observed with both continuous and pulse feeding strategies. Significant shifts in plasmatic magnesium, calcium, and chloride levels were recorded, while immunological parameters remained unaffected.

In this continuation study, juvenile rainbow trout will be challenged with the key salmonid pathogen *A*. *salmonicida* subsp. *salmonicida* strain 89409 to verify the effects of the probiotic under infection pressure. The study will assess the impact of two supplementation strategies on fish survival, gut microbiota, and health status based on haematological, plasma biochemical and immunological variables. As economic burden could be a critical factor for fish producers, cyclic supplementation will be introduced alongside continuous supplementation. While the beneficial effects of lactic acid bacteria as probiotics in controlling furunculosis have previously been documented (e.g., Irianto and Austin 2002), the present study is novel in that it makes a comparative assessment of two distinct probiotic feeding strategies.

## MATERIAL AND METHODS

### Experimental design and fish rearing conditions

In the previous study by Mikulikova et al. (2025), an all-female population of juvenile rainbow trout was fed for 7 weeks with feed enriched with *L.* *plantarum* R2 Biocenol^TM^ (CCM 8674) bacterial cells (group P). A second group received the same feed for four weeks, followed by three weeks of control feed (group PC), while a control group (group C) received feed without incorporated lactobacilli. In all cases, the fish were fed at a rate of 2.68% of their total body weight, and were reweighed twice during the experiment to adjust the feeding rate based on their weight gain. After seven weeks, the mean length of fish in group C was 256.65 ± 12.4 mm, 258.28 ± 10.95 mm in group PC, and 258.97 ± 12.83 mm in group P, while the mean body weight was 316.03 ± 44.32 g, 314.35 ± 40.67 g, and 319.53 ± 45.97 g, respectively.

**Figure 1:**
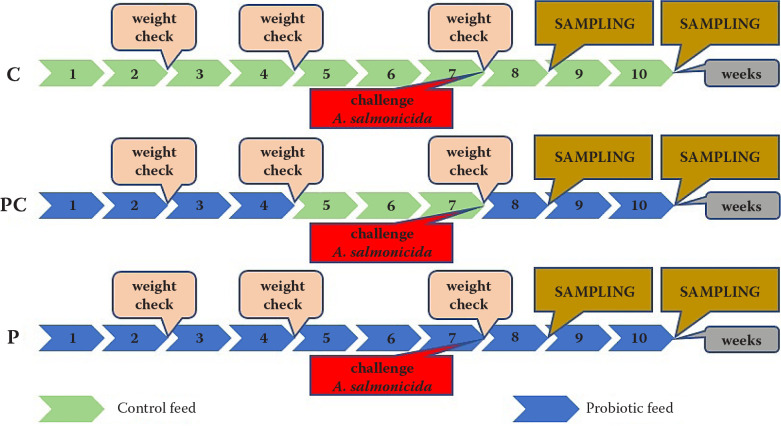
Experimental timeline C = control; P = probiotic-enriched feed; PC = probiotic/control feed

For this next stage of the study, 300 fish (100 fish per treatment group) were transported to the experimental facility at the University of Veterinary Sciences in Brno. Upon arrival, the fish (each group kept separately) were challenged with 7.33 × 10^5^ CFU/ml of *Aeromonas salmonicida* subsp. *salmonicida* (hereafter *Ass*) strain 89409 via a 30-minute bath exposure (Figure 1).

Subsequently, the fish were placed into six 1 000 l tanks (50 fish/tank, i.e., two tanks per treatment), each representing an individual recirculation aquaculture system equipped with a drop-in biofilter. Throughout the experiment, the photoperiod was maintained at 14 h of light and 10 h of darkness. The feeding regime was as follows: the cyclic supplementation group (PC) and the continuously supplemented group (P) were given feed enriched with *L.* *plantarum* R2 Biocenol^TM^ (CCM 8674) bacterial cells, which were incorporated into a starch hydrogel layer (for details see Mikulikova et al. 2025), while the control group (C) received commercial pellets (EFICO Enviro 921, 3 mm; BioMar, Brande*,* Denmark) with a hydrogel coating with no bacterial cells incorporated. The viable count of *L. plantarum* was approximately 10^8^ CFU/g (MRS agar, anaerobic incubation at 37 °C for 48 hours). All feeds were stored at 4 °C until use. The fish received either probiotic or control feed, with no mixing between diets.

The health status of the fish was monitored twice daily; any deceased fish were removed, dissected, and a bacteriological culture of the spleen was performed on blood agar.

Cumulative mortality was then calculated. In addition, two samplings were taken from all tanks, the first one week after bacterial challenge, and the second three weeks post-challenge (when the experiment ended). In each case, the number of fish sampled depended on the mortality rate in each tank. As with fish mortalities, the sampled fish were dissected for examination, and a spleen cultivation was carried out.

Water quality parameters (temperature, oxygen saturation, and pH) were monitored twice daily in all tanks and remained within optimal ranges for rainbow trout welfare. Oxygen saturation was over 80% in all tanks, with temperatures of 16.88 ± 0.52 °C, 16.86 ± 0.62 °C, and 16.43 ± 0.45 °C, and pH values of 7.99 ± 0.14, 7.91 ± 0.14, and 7.99 ± 0.12, in the C, PC, and P groups, respectively.

### Blood examination for haematological, biochemical and immune parameters

Blood samples (2 ml) were collected from the caudal vessels using heparinised syringes (50 IU heparin/ml; Zentiva, Prague, Czech Republic). The fish were then euthanised by severing their vertebral columns at the skull base. The following basic haematological parameters were determined according to Svobodova et al. (2012): red blood cell count, white blood cell count, haematocrit, haemoglobin, mean cell corpuscular volume, mean cell corpuscular haemoglobin, mean cell haemoglobin concentration, and lymphocyte-to-phagocyte ratio.

Phagocytic activity was assessed as respiratory burst activity using luminol-enhanced chemiluminescence (Sigma-Aldrich, Steinheim am Albuch, Germany), as described by Papezikova et al. (2016). Chemi-luminescence was recorded for 90 min using a Cytation 3M reader (BioTek, Winooski, VT, USA), with the results expressed as peak time and total intensity (integral of the curve).

Following centrifugation at 800 *g* for 10 min at 4 °C, the blood plasma was analysed for the following biochemical indices: ferric reducing ability of plasma (FRAP), ceruloplasmin activity, biopterin, IgM, interleukin-10 (IL-10) and C-reactive protein. The following plasma biochemical indices were measured photometrically using a Konelab 20i biochemical analyser with commercial kits (Biovendor, Brno, Czech Republic): total protein, albumin, ammonia, triglycerides, cholesterol, creatinine, calcium, inorganic phosphorus, chloride, magnesium, glucose, lactate, creatine kinase, aspartate aminotransferase, alkaline phosphatase, alanine aminotransferase and lactate dehydrogenase. FRAP was determined according to Benzie and Strain (1996), as modified by Haluzova et al. (2010). Ceruloplasmin activity was evaluated using the *p*-phenylenediamine method of Ceron and Martinez-Subiela (2004). Biopterin concentration was determined by high-performance liquid chromatography, as per Carru et al. (2004), using a Dionex Ultimate 3000 system (Thermo Fisher Scientific, Waltham, MA, USA) with a Zorbax Bonus-RP C18 column (Agilent Technologies, Santa Clara, CA, USA). IgM, IL-10, and C-reactive protein levels were measured using commercial ELISA kits (MyBioSource, San Diego, CA, USA), following the manufacturer’s instructions.

The experiment was performed in compliance with the Czech law on protection of animals against cruelty, as approved by the Czech Ministry of Education, Youth and Sports (Permit No. MSMT-5936/2022-4)

### Statistical analysis of non-micro-biome data

All statistical analyses were conducted using Unistat for Excel v6.5 (2017; Unistat Ltd., UK). Data normality and variance homogeneity were assessed using the Shapiro–Wilk and Levene’s tests. Normally distributed data were then analysed using one-way ANOVA with Tukey’s HSD post-hoc test, and non-normal data by Kruskal-Wallis ANOVA with multiple median comparisons. Comparisons were made between groups at the same time point, with statistical significance set at *P* < 0.05. Results are expressed as mean ± standard deviation (SD). Differences in cumulative mortality between groups were assessed using chi-squared tests based on contingency tables. Overall group differences were evaluated using Pearson’s chi-squared test (3 × 2 tables), followed by post-hoc pairwise comparisons (2 × 2 tables) using Yates’ continuity correction, with a *P*-value of <0.05 considered statistically significant. Any parameters that did not have enough samples in any of the variants were excluded from the statistical evaluation.

### Microbiome analysis

Gut microbial communities were assessed using five fish per treatment group, sampled three weeks post-challenge. The distal intestine (10–12 cm) was removed and frozen immediately at –80 °C for subsequent processing. After collecting 100 μg of intestinal contents, DNA was isolated using an EZNA Soil Kit (Omega, Norcross, GA, USA) and stored at –20 °C. The 16S rRNA gene was first amplified with a 16S Barcoding Kit and then sequenced on a MinION Mk1B device (Oxford Nanopore Technologies plc, Oxford, UK). Low-quality reads (Phred <7) were removed before taxonomic classification was performed using the RDP16S_v18 database. After rarefaction, total sum scaling normalisation and removal of mitochondrial and chloroplast reads, the resulting operational taxonomic unit (OTU) table was analysed in Microbiome Analyst. Alpha (Shannon, Chao1, ANOVA) and beta [principal component analysis (PCoA), PERMANOVA] diversity, correlation, and pattern analyses were then conducted at the genus level.

## RESULTS AND DISCUSSION

### Mortality and pathological findings

**Figure 2:**
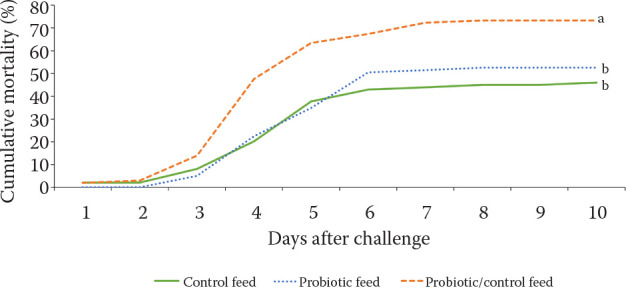
Cumulative mortality in rainbow trout following challenge with *Aeromonas salmonicida* ^a,b^Different letters indicate significant differences between groups at day 10 post challenge with *A.* *salmonicida* (*P* < 0.05)

The first mortality occurred on the day after the bacterial challenge, with further mortalities peaking after four to six days and only sporadic deaths recorded thereafter (Figure 2). Our experimental challenge conditions were designed to simulate an acute infection, which mainly progresses during the first week after exposure (Moreau et al. 2023). Real-time bioluminescence imaging of *A. salmonicida* infection in rainbow trout has demonstrated that colonisation typically begins at the dorsal and pectoral fins and gills, subsequently spreading to the internal organs (digestive tract, spleen, kidney), and that bacteria are shed through the anal opening (Bartkova et al. 2017). Our pathological examination of affected fish revealed lesions characteristic of furunculosis, including skin erosion, congestion of the caudal intestine with yellowish mucus or haemorrhagic content, splenomegaly, and marbling of the kidneys. In addition, haemorrhagic fluid was present in the body cavity and multiple haemorrhages were observed on the skin, gills, peritoneum, adipose tissue, and swim bladder. Abscess formation in the musculature was detected in a few specimens from day-5 post-challenge onwards. Bacterial cultures of the spleen confirmed the presence of *Ass* in all deceased fish. In contrast, no visible signs of the disease were observed during post-mortem examination of fish sampled one week and three weeks post-challenge, with almost all spleen cultures from these fish proving negative (except for two individuals sampled one week post-challenge).

Despite expectations based on previous *in vitro* tests (Cingelova Maruscakova et al. 2021), our *in vivo* experiment did not result in an enhanced survival rate among challenged fish under either supplementation regime tested, compared with the control group. Moreover, the cumulative mortality of fish receiving probiotics under cyclic feeding (PC: 73%) was significantly higher (*P* < 0.05) than that in the continuous probiotic (P: 52%) or control (C: 46%) groups. It can be hypothesised that the fish’s resistance to bacterial infection may have been adversely affected by fluctuations in the composition of their gut microbiota induced by intermittent dietary changes.

### Haematological, plasma biochemical and immune indices

One week after the challenge, no significant differences were observed in most of the parameters tested (Tables 1, 2 and 3). Shieh and Maclean (1976) investigated changes in haemoglobin concentration and selected biochemical parameters in the serum of brook trout (*Salvelinus fontinalis*) three days after intramuscular inoculation with *A. salmonicida*. Of the parameters also measured in our study, they observed significant increases in urea, creatinine, ammonia, and glucose levels, alongside significant decreases in haemoglobin, total protein, triglycerides, cholesterol, and inorganic phosphorus, compared to healthy controls. In our study, all groups were infected, and these parameters did not differ significantly between them.

**Table 1:** Haematological parameters (mean ± SD) for rainbow trout following challenge with *Aeromonas salmonicida*

Parameter	Unit	Sampling time post-challenge (weeks)	Control (*n* = 15−16)	Probiotic/control feed (*n* = 5−14)	Probiotic-enriched feed (*n* = 15−16)
RBC	10^12^/l	1	1.30 ± 0.33	1.49 ± 0.36	1.24 ± 0.26
		3	1.21 ± 0.26	1.13 ± 0.08	1.15 ± 0.14
					
Hb	g/l	1	84.78 ± 18.00	89.50 ± 14.04	88.71 ± 14.00
		3	93.46 ± 15.81	91.57 ± 7.40	90.97 ± 12.08
					
Ht	l/l	1	0.42 ± 0.09	0.41 ± 0.05	0.41 ± 0.07
		3	0.42 ± 0.05	0.43 ± 0.02	0.41 ± 0.06
					
MCV	fl	1	330.07 ± 48.00^ab^	289.91 ± 56.14^b^	335.89 ± 44.58^a^
		3	354.35 ± 49.16	378.39 ± 12.27	354.39 ± 35.65
					
MCH	pg	1	66.74 ± 10.10^ab^	62.61 ± 12.86^b^	72.49 ± 7.86^a^
		3	78.12 ± 7.33	81.01 ± 2.02	79.60 ± 7.99
					
MCHC	g/l	1	202.53 ± 13.86	215.69 ± 16.91	217.46 ± 22.67
		3	222.37 ± 19.01	214.27 ± 8.70	225.12 ± 15.58
					
WBC	10^9^/l	1	44.27 ± 14.59	28.71 ± 12.10	32.50 ± 12.48
		3	18.00 ± 5.18	18.60 ± 7.16	16.47 ± 3.96
					
Lymphocytes	%	1	83.38 ± 5.08	82.86 ± 5.96	83.88 ± 7.26
		3	83.00 ± 6.65^ab^	86.60 ± 9.45^b^	87.60 ± 8.83^a^
					
Lymphocytes	10^9^/l	1	34.82 ± 15.21^a^	23.57 ± 9.65^b^	27.53 ± 11.41^ab^
		3	14.83 ± 4.00	15.62 ± 3.91	14.32 ± 3.32
					
Phagocytes	%	1	16.63 ± 5.08	17.14 ± 5.96	16.13 ± 7.26
		3	17.00 ± 6.65^a^	13.40 ± 9.45^b^	12.40 ± 8.83^ab^
					
Phagocytes	10^9^/l	1	6.87 ± 3.04	5.15 ± 3.08	4.97 ± 2.55
		3	3.17 ± 1.65^a^	2.98 ± 3.42^b^	2.15 ± 1.84^ab^

^a,b^Different superscripts along a row denote significance (*P* < 0.05)

Hb = haemoglobin; Ht = haematocrit; MCV = mean corpuscular volume; MCH = mean corpuscular haemoglobin; MCHC = mean corpuscular haemoglobin concentration; RBC = red blood cell count; WBC = white blood cell count

**Table 2:** Plasma biochemical indices (mean ± SD) for rainbow trout following challenge with *Aeromonas salmonicida*

Parameter	Unit	Sampling time post-challenge (weeks)	Control (*n* = 8−14)	Probiotic/control feed (*n* = 5−11)	Probiotic-enriched feed (*n* = 7−15)
Total protein	g/l	1	35.10 ± 5.82	36.84 ± 5.41	34.29 ± 3.01
		3	38.64** ± **4.17^a^	39.11** ± **8.93^b^	39.63** ± **6.20^ab^
					
Albumin	g/l	1	17.33 ± 3.65	17.65 ± 3.01	16.03 ± 2.18
ALT	μkat/l	1	0.58 ± 0.48	0.49 ± 0.21	0.55 ± 0.40
AST	μkat/l	1	9.01 ± 4.67	6.46 ± 2.81	7.96 ± 4.63
ALP	μkat/l	1	1.04 ± 0.43	0.88 ± 0.39	0.93 ± 0.41
CK	μkat/l	1	89.44 ± 72.09	52.18 ± 30.24	74.25 ± 34.86
LDH	μkat/l	1	19.21 ± 11.23	29.72 ± 29.65	24.92 ± 17.16
Ammonia	μmol/l	1	873.40 ± 201.20	902.50 ± 91.14	952.42 ± 165.36
Creatinin	μmol/l	1	27.57 ± 5.74	29.89 ± 4.44	27.38 ± 5.40
Triglycerides	mmol/l	1	1.28 ± 0.36	1.22 ± 0.30	1.34 ± 0.52
Cholesterol	mmol/l	1	7.69 ± 1.09	7.87 ± 1.60	7.56 ± 1.76
					
Glucose	mmol/l	1	3.52 ± 1.24	3.78 ± 1.11	3.32 ± 0.69
		3	5.31 ± 1.51	4.58 ± 1.19	5.65 ± 1.51
					
Lactate	mmol/l	1	5.01 ± 1.96	4.89 ± 2.60	4.12 ± 0.86
Chloride	mmol/l	1	123.62 ± 4.60	125.62 ± 4.33	125.21 ± 2.00
					
Phosphorus	mmol/l	1	5.78 ± 1.62	5.94 ± 1.04	5.97 ± 1.02
		3	3.49 ± 0.66	4.04 ± 0.43	3.79 ± 0.94
					
Magnesium	mmol/l	1	0.98 ± 0.24	1.14 ± 0.12	1.03 ± 0.13
Calcium	mmol/l	1	2.73 ± 0.32	2.86 ± 0.32	2.86 ± 0.22

^a,b^Different superscripts within a row denote significance (*P* < 0.05)

ALT = alanine aminotransferase; ALP = alkaline phosphatase; AST = aspartate aminotransferase; CK = creatine kinase; LDH = lactate dehydrogenase

One week after bacterial challenge, reductions in lymphocyte count and phagocyte activity were noted in PC fish; however, the decrease in phagocyte activity was not evident when expressed per 1 000 phagocytes (Table 1). This suggests that the respiratory burst in individual phagocytic cells was probably not significantly impaired, and that the decrease in total phagocyte count was non-significant due to the high variability between fish. However, in an experiment on Atlantic cod (*Gadus morhua*), genes associated with reactive oxygen species production were found to be downregulated (Soto-Davila et al. 2019). This suggests that *A. salmonicida* may use its virulence factors to modulate and inhibit macrophage antibacterial responses.

Three weeks after the challenge, the PC group showed a significant decrease in total phagocyte count and the proportion of phagocytes among white blood cells (Table 1). Compared with the control, IgM levels were significantly lower in both the P and PC groups, whereas reductions in interleukin-10 and increases in total protein were observed only in the PC group (Tables 2 and 3). The more severe course of the disease in PC fish, as confirmed by the significantly higher mortality rate, was presumably accompanied by an immunosuppressive effect of the pathogen, along with exhaustion of phagocytosis and mobilisation of phagocytes to infected tissues. The production of antibodies in fish is affected by various factors, including water temperature, season or photoperiod, and the amount of antigen (Romstad et al. 2013; Montero et al. 2022). In teleost fish, IgM is the main circulating immunoglobulin and exists in monomeric and tetrameric isoforms. Following bacterial infection, its serum levels increase, and a shift from the monomeric to the tetrameric form occurs (Costa et al. 2012). The presence of specific anti-*A. salmonicida* IgM antibodies were observed in the serum of rainbow trout two weeks after they were injected with inactivated *Ass* (Montero et al. 2022), whereas anti-*Ass* antibodies were observed four weeks after experimental challenge via immersion bath (Moreau et al. 2023). The latter study also revealed that the mortality rate following immersion exposure depended on contact time. However, in our experiment, the bath duration was identical for all fish. In sablefish (*Anoplopoma fimbriata*) vaccinated against *A.* *salmonicida*, total IgM titres peaked at six to eight weeks post-immunisation (Vasquez et al. 2020), whereas in our study, lower levels of IgM in P and PC fish three weeks after challenge suggested a higher degree of immunosuppression due to experimental infection.

**Table 3:** Immunological indices (mean ± SD) for rainbow trout following challenge with *Aeromonas salmonicida*

Parameter	Unit	Sampling time post-challenge (weeks)	Control (*n* = 7−16)	Probiotic/control feed (*n* = 5−14)	Probiotic-enriched feed (*n* = 7−16)
FRAP	Fe^2+^ eq. mmol/l	1	674.88 ± 164.56	614.00 ± 165.55	653.45 ± 91.52
					
Ceruloplasmin	∆ absorb/min ×10 000	1	196.54 ± 53.04	234.10 ± 46.27	201.31 ± 30.04
		3	209.76 ± 42.11	260.10 ± 37.52	219.48 ± 46.14
					
CRP	mg/ml	1	10.76 ± 5.57	13.81 ± 7.61	11.01 ± 5.19
		3	19.99 ± 12.91	23.17 ± 7.15	26.28 ± 12.68
					
IgM	mg/ml	1	304.21 ± 215.24	395.18 ± 168.54	289.21 ± 118.92
		3	365.33 ± 137.82^a^	303.56 ± 196.47^b^	253.19 ± 178.41^b^
					
IL-10	pg/ml	1	7.81 ± 1.26	10.10 ± 4.46	7.93 ± 2.47
		3	10.15 ± 2.73^a^	8.10 ± 0.52^b^	10.47 ± 3.29^a^
					
Biopterin	nmol/l	1	15.73 ± 2.41	15.55 ± 2.50	17.12 ± 5.09
		3	14.60 ± 2.20	14.47 ± 3.18	15.25 ± 2.70
					
PA peak time	min	1	59.25 ± 13.96	60.86 ± 11.13	68.25 ± 11.41
		3	49.60 ± 9.61	54.00 ± 15.73	51.80 ± 9.03
					
PA int.	RLU.min	1	255 766.69 ± 156 070.95^a^	205 523.46 ± 161 805.96^b^	203 837.06 ± 141 911.55^ab^
		3	313 632.40 ± 239 145.11	265 353.90 ± 199 677.36	280 065.90 ± 185 257.39
					
PA int./1 000 fag	RLU.min	1	1 838.98 ± 911.60	2 155.58 ± 1 983.33	2 139.21 ± 955.44
		3	4 696.98 ± 2 313.17^ab^	6 644.72 ± 6 227.30^b^	8 482.26 ± 7 035.18^a^
					
PA peak/1 000 fag	RLU	1	43.32 ± 21.53	49.10 ± 45.86	49.65 ± 19.89
		3	104.53 ± 45.28^ab^	144.17 ± 136.48^b^	191.67 ± 151.02^a^

^a,b^Different superscripts within a row denote significance (*P* < 0.05)

CRP = C-reactive protein; FRAP = ferric reducing ability of plasma; IgM = immunoglobulin M; IL-10 = interleukin-10; int. = intensity; PA = phagocytic activity; RLU = relative light unit

In fish, changes in total plasma protein levels after bacterial infection may indicate physiological adaptations, given that this parameter encompasses a wide variety of plasma proteins involved in processes such as osmotic regulation, nutrient transport, acute-phase responses, and adaptive immune responses. As IgM levels decreased concurrently in the same fish, the increase in total plasma protein may indicate an increase in acute-phase proteins. Unfortunately, a detailed characterisation of the plasma protein profile was not performed in this study. Interleukin-10 is a pleiotropic cytokine produced by various immune cell populations, such as macrophages, T cells, B cells, and natural killer cells, and it regulates their functions during the immune response. In teleost fish, IL-10 is the main anti-inflammatory cytokine, whose production can be triggered by conserved microbial structures (Harun et al. 2011).

### Microbiome

Sequencing of caudal intestine content revealed 1 219 884 total reads, 81 326 average reads per sample and a minimum count of 12 549 reads. After removing singletons, 132 OTUs were identified for subsequent analysis. From these, 77 low-abundance OTUs (minimum count four in 20% of samples) and six low-variance OTUs (10% removal based on inter-quartile range) were excluded from further statistical analyses. After filtering, therefore, 49 OTUs remained for analysis of gut microbial community composition. Due to differences in the number of reads between libraries, the data were rarefied to the minimum library size (12 549 reads) and normalised using total sum scaling.

**Figure 3:**
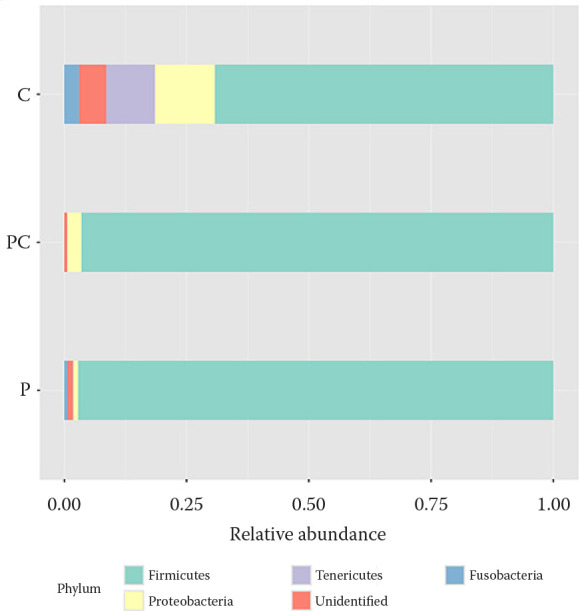
Relative abundance of phyla in rainbow trout gut content three weeks after challenge with *Aeromonas salmonicida* (i.e., three weeks after change of diet in the PC group C = control; P = probiotic-enriched feed; PC = probiotic/control feed

At the phylum level, Firmicutes (69.0, 96.5 and 97.2%), Proteobacteria (12.2, 2.9 and 1.0%), Tenericutes (10.0, 0.03 and 0.1%) and Fusobacteria (3.0, 0.0 and 0.6%) were recorded in faecal samples from the caudal part of the intestine in groups C, PC and P, respectively (Figure 3).

**Figure 4:**
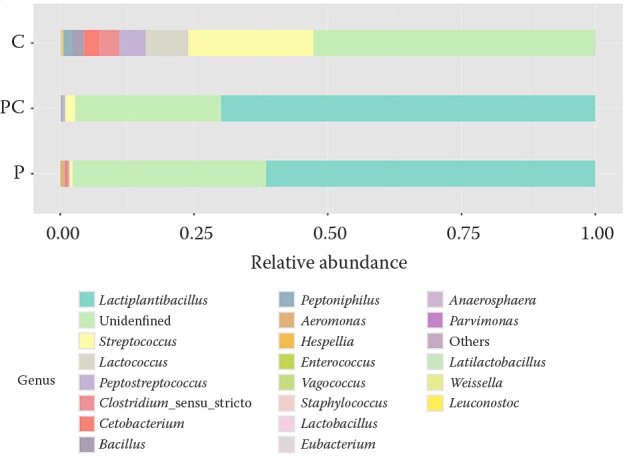
Relative abundance of genera in rainbow trout gut content three weeks after challenge with *Aeromonas salmonicida* (i.e., three weeks after change of diet in PC group C = control; P = probiotic-enriched feed; PC = probiotic/control feed

At the genus level, gut analysis revealed the presence of 20 genera, with *Streptococcus* (23.4%), *Lactococcus* (8.0%), and* Peptostreptococcus* (4.9%) most prevalent in group C, and *Lactiplantibacillus* dominant in the PC (70.0%) and P (61.5%) groups (Figure 4). In the PC and P groups, the abundance of five genera detected in C fish did not exceed 0.000 0%.

**Figure 5:**
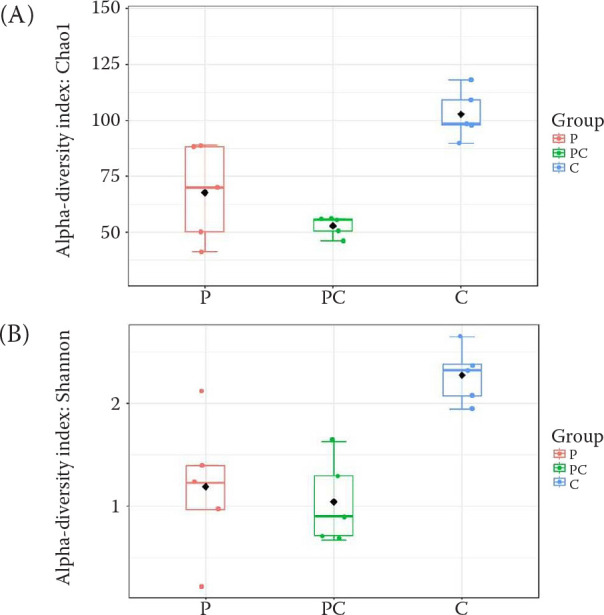
Distal gut microbial community Alpha diversity (A) Chao1 index (*P* < 0.001, ANOVA), highly significant difference between the C and PC groups (*P* < 0.001) and a significant difference between the C and P group (*P* < 0.05); (B) Shannon index (*P* < 0.01, ANOVA), significant difference between the C and P groups (*P* < 0.05) and a highly significant difference between the C and PC groups (*P* < 0.001) in rainbow trout three weeks after challenge with *Aeromonas salmonicida* (i.e., three weeks after change of diet in the PC group bottom/top line of box = lower/upper quartile; lower/upper whisker = lower/upper adjacent value; middle line of box = median; C = control; P = probiotic-enriched feed; PC = probiotic/control feed; rhombus = mean

**Figure 6:**
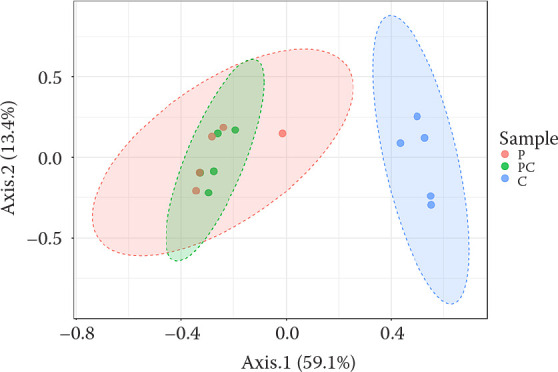
Distal gut microbial community Beta diversity (PCoA based on Bray–Curtis distance, *P* < 0.01, PERMANOVA), significant differences in microbial community composition between the C and PC groups (*P* < 0.01) and the C and P groups (*P* < 0.05) in rainbow trout three weeks after challenge with *Aeromonas salmonicida* (i.e., three weeks after change of diet in the PC group C = control; P = probiotic-enriched feed; PC = probiotic/control feed

**Figure 7:**
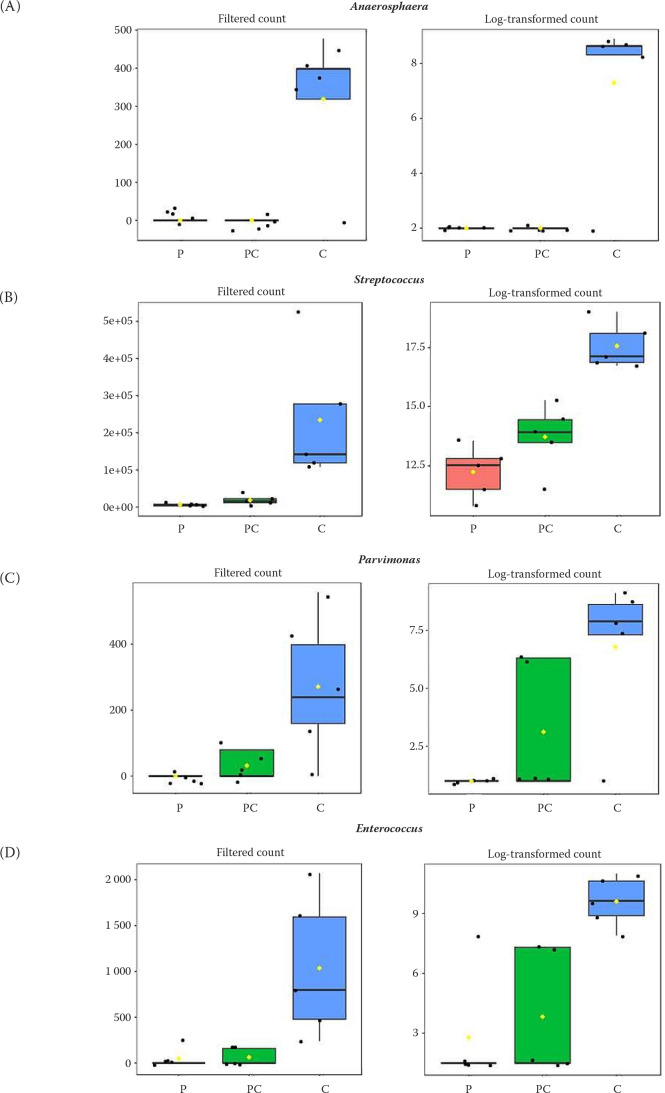

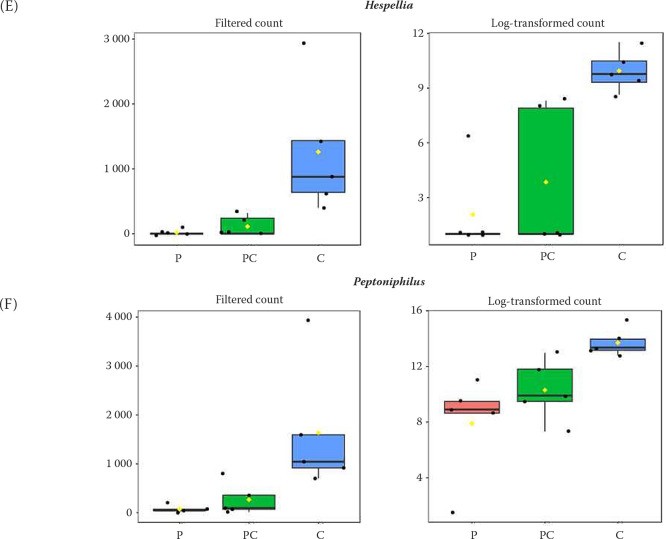
Correlation patterns (filtered count and log-transformed count, Pearson *r*, *P* < 0.01) in genera (A) *Anaerosphera*, (B) *Streptococcus*, (C) *Parvimonas*, (D) *Enterococcus*, (E) *Hespelia* and (F) *Peptoniphilus* bottom/top line of box = lower/upper quartile; lower/upper whisker = lower/upper adjacent value; middle line of box = median; rhombus = mean; C = control; P = probiotic-enriched feed; PC = probiotic/control feed

Alpha diversity (Figure 5) analysis of bacterial taxon abundance in the three test groups (C, PC and P) indicated a highly significant difference in the Chao1 index (*P* < 0.001, ANOVA), with a highly significant difference between the C and PC groups (*P* < 0.001) and a significant difference between the C and P group (*P* < 0.05). The Shannon index (*P* < 0.01, ANOVA) indicated a significant difference between the C and P groups (*P* < 0.05) and a highly significant difference between the C and PC groups (*P* < 0.001). Beta diversity (Figure 6), as determined by a PCoA based on Bray-Curtis distance (*P* < 0.01, PERMANOVA), revealed significant differences in microbial community composition between the C and PC groups (*P* < 0.01) and the C and P groups (*P* < 0.05). A highly significant correlation pattern (*P* < 0.01) was observed for the abundance of the genera *Anaerosphera*, *Streptococcus*, *Parvimonas*, *Enterococcus*, *Hespelia,* and *Peptoniphilus* (Figure 7).

The best-known functional roles of microbiota are those related to digestion and immune regulation. A stable and diverse gut microbiome helps protect fish against harmful bacteria, which remain a major cause of mortality in aquaculture. The ability of bacteria to attach to epithelial cells is considered a key factor in determining how effectively they can act within the host. There is evidence to suggest that the ability to form biofilms is closely linked to successful colonisation and the functional performance of the gut microbiota. Biofilm formation enables bacteria to withstand the challenging conditions of the gastrointestinal environment, and this phenomenon has been observed in both pathogenic and probiotic species (Talwar et al. 2018).

As the fish collected three weeks after being challenged with *Ass* for gut content microbial analysis represent those that successfully withstood infection, it can be assumed that these individuals adapted most effectively to the dietary modifications during the infection. This likely explains why no significant differences in gut microbiome composition were detected between the P and PC groups. Another reason is that three weeks may have been a sufficiently long period for the gut microbiota to adjust to the supplementation. Although a reduction in gut microbial diversity was observed in both experimental groups, the only difference between them was the diet they received prior to challenge. This factor may be considered a key determinant of the observed differences in mortality following *Ass* infection.

The findings demonstrate that supplementation with *L. plantarum* R2 Biocenol^TM^ does not improve the survival rate of rainbow trout following *A. salmonicida* infection. Not only did the observed outcomes diverge from expectations based on previous *in vitro* assays, but the feeding regimen involving cyclic supplementation (PC group) also resulted in significantly higher mortality. These findings highlight the importance of a stable and well-balanced gut microbiome for maintaining effective immune responses in fish and indicate that inappropriate modulation of the microbiota may have detrimental consequences. From an applied perspective, these results do not support the use of *L. plantarum* R2 Biocenol^TM^ as a strategy to enhance resistance to *A. salmonicida* infection in rainbow trout. At the same time, the study provides valuable *in vivo* evidence on the limitations of translating *in vitro* probiotic effects into practice. Future research should explore alternative approaches to microbiome modulation, including dietary composition, prebiotic or synbiotic strategies, and management-related factors influencing microbiome stability and host immune responses.
